# Genetic Determinism Exists for the Global DNA Methylation Rate in Sheep

**DOI:** 10.3389/fgene.2020.616960

**Published:** 2020-12-23

**Authors:** Dominique Hazard, Florence Plisson-Petit, Carole Moreno-Romieux, Stéphane Fabre, Laurence Drouilhet

**Affiliations:** GenPhySE, Université de Toulouse, INRAE, ENVT, Castanet Tolosan, France

**Keywords:** epigenetics, blood, heritability, genetic correlation, QTL

## Abstract

Recent studies showed that epigenetic marks, including DNA methylation, influence production and adaptive traits in plants and animals. So far, most studies dealing with genetics and epigenetics considered DNA methylation sites independently. However, the genetic basis of the global DNA methylation rate (GDMR) remains unknown. The main objective of the present study was to investigate genetic determinism of GDMR in sheep. The experiment was conducted on 1,047 Romane sheep allocated into 10 half-sib families. After weaning, all the lambs were phenotyped for global GDMR in blood as well as for production and adaptive traits. GDMR was measured by LUminometric Methylation Analysis (LUMA) using a pyrosequencing approach. Association analyses were conducted on some of the lambs (*n* = 775) genotyped by using the Illumina OvineSNP50 BeadChip. Blood GDMR varied among the animals (average 70.7 ± 6.0%). Female lambs had significantly higher GDMR than male lambs. Inter-individual variability of blood GDMR had an additive genetic component and heritability was moderate (*h*^2^ = 0.20 ± 0.05). No significant genetic correlation was found between GDMR and growth or carcass traits, birthcoat, or social behaviors. Association analyses revealed 28 QTLs associated with blood GDMR. Seven genomic regions on chromosomes 1, 5, 11, 17, 24, and 26 were of most interest due to either high significant associations with GDMR or to the relevance of genes located close to the QTLs. QTL effects were moderate. Genomic regions associated with GDMR harbored several genes not yet described as being involved in DNA methylation, but some are already known to play an active role in gene expression. In addition, some candidate genes, *CHD1*, *NCO3A, KDM8, KAT7*, and *KAT6A* have previously been described to be involved in epigenetic modifications. In conclusion, the results of the present study indicate that blood GDMR in domestic sheep is under polygenic influence and provide new insights into DNA methylation genetic determinism.

## Introduction

Genetic selection in several livestock species has recently progressed to include heritable DNA polymorphisms (i.e., genomic selection) for the improvement of production traits (Goddard et al., 2010). However, recent studies also showed that non-genetic information responsible for phenotypic differences between animals can also be inherited across generations (Johannes et al., 2008; Danchin et al., 2011). One such non-genetic inherited piece of information concerns epigenetic marks. Epigenetic modifications include biochemical modifications of DNA (methylation) or proteins tied to DNA (methylation and acetylation of histone proteins). These epigenetics processes act in an interrelated way to influence gene expression and hence phenotype in response to environmental conditions, thereby demonstrating that the eukaryotic genome can respond dynamically to changes in the environment to which every individual is exposed (Geiman and Robertson, 2002).

One of the key mechanisms in the regulation of gene expression in mammals is cytosine methylation at CpG dinucleotides, the most common DNA modification in eukaryotes. The importance of epigenetics in mammals and plants has been demonstrated in several studies and epigenetics contribute to the inheritance of traits of interest. In plants, DNA methylation in several differentially methylated regions was shown to explain 60–90% of the heritability of complex traits (Cortijo et al., 2014). In humans, DNA methylation explained 7–11% of the body mass index while SNP explained 5–8% of the same trait (Dick et al., 2014). In livestock, current studies aim to identify epi-loci underlying traits of agronomical interest. For instance, in sheep, genome-wide analysis of DNA methylation profiles has already revealed numerous epi-loci associated with prolificacy or body size (Cao et al., 2015; Zhang et al., 2017). Epigenetic sources of inheritance are not currently taken into consideration in selection strategies in livestock production but these studies could help identify the origin of variability of production traits and later on, improve genetic progress.

Taking into account variations in epigenetic marks may enable better modeling of phenotypic variation and improving the precision of genetic evaluation of traits and breeds. In addition, taking epigenetic events into account may help not only understand but also improve adaptive processes in both plants and mammals. Particular epigenetic recombinant inbred lines of *Arabidopsis thaliana* showed highly variable DNA methylation, while being genetically very similar (Latzel et al., 2013). Using these plant model lines, it was demonstrated an important role of epigenetics in adaptive process facing saline conditions for example (Kooke et al., 2015). Moreover, evolutionary studies showed that epigenetic inheritance may be important for natural selection and transmission of advantageous traits, for a review (see Donohue, 2014). Recent advances in epigenetic studies in natural populations addressed how epigenetic inheritance may influence adaptive evolution by focusing on epigenetic stability and inheritance itself as a potentially evolving trait (Herman et al., 2014; Schlichting and Wund, 2014). These articles brought into focus the genetic and ecological basis of epigenetic stability and raised a number of questions including “is the stability of epigenetic marks heritable?”

Turck and Coupland (2014) showed that DNA methylation has the potential to be mitotically and meiotically stable, whereas histone modification is involved in environmentally induced epigenetic regulation that may be reversible. These authors hypothesized that both the degree of DNA methylation and the stability of environmentally induced changes in gene expression via histone modification are associated with changes in the DNA sequence that can cause heritable variation in epigenetic regulation. New scientific questions arise concerning the relationship between genetics and epigenetics as part of the improvement of livestock species and their adaptation to changing production systems. In the present study, we used a complementary approach to those used in existing studies by considering the global methylation of genomic DNA as a novel quantitative phenotype to provide essential information on the processes involved in epigenetic inheritance. DNA methylation in differentially methylated regions representing a very small proportion of genomic DNA global methylation, we hypothesized that the stable fraction of DNA methylation is genetically determined and can be inherited across generations. To test this hypothesis, we quantified the global DNA methylation rate in blood samples collected from domestic sheep. Sheep not only has the advantage of being a species of agronomical interest, but also represents an interesting animal model to study adaptive processes. The aim of the present study was to estimate the genetic component of the global DNA methylation rate and to identify the genomic loci underlying the phenotype.

## Materials and Methods

### Animals and Management

The experimental animals were Romane lambs, a fixed crossbreed between Romanov x Berrichon du Cher (Ricordeau et al., 1992). Data collected from a total of 1,047 male and female lambs over a period of 5 years were used in this study. The lambs originated from 10 rams, each ram was used for only 1 year. All the animals were born in April, identified at birth using electronic ear tags, and reared outdoors with their dams under extensive conditions in the experimental “la Fage” farm on 280 ha of rangeland (Causse du Larzac, Roquefort sur Soulzon, South of France). The farming system, management and environment characteristics are detailed in Gonzalez-Garcia et al. (2014). The lambs were weighed regularly from lambing to weaning to estimate average daily gain. All the lambs were weaned at 85 ± 4 days of age. Two weeks after weaning, experimental lambs were individually exposed to two behavioral tests. Tests of each animal were all performed on the same day, with a total of around 35 lambs tested per day on 10–11 consecutive test-days per year. After the behavioral tests (i.e., approximately 1 month after weaning), lambs were blood sampled for genotyping and determination of the global DNA methylation rate.

### Global DNA Methylation Rate

The global DNA methylation rate (GDMR) was measured from whole blood samples taken from sheep. Genomic DNA was extracted from the blood samples using a high-salt extraction method (Roussot et al., 2003). Methylation analyses were performed using the LUMA assay (Karimi et al., 2006a,b). Briefly, this method relies on the use of methylation-sensitive and –insensitive restriction endonucleases: *Hpa*II and *Msp*I, respectively. The target sequence for both enzymes is CCGG, *Hpa*II is not able to cut if the internal cytosine is methylated (C^*m*^CGG), whereas *Msp*I cuts the restriction site whatever the methylation status. Moreover, *Eco*RI (recognition site: GAATTC) was used as an internal control for normalization of DNA amount. For each sample, DNA was independently digested by *Eco*RI + *Hpa*II and *Eco*RI + *Msp*I restriction enzymes (New England Biolabs) and then analyzed using a Q24 Pyromark sequencer and Q24 1.0.10 software (Qiagen). The dispensation order for nucleotides was GTGTCACATGTGTG. Methylation rate was calculated from peak heights as [1 − [(*Hpa*II(G)/ *Eco*RI_Hpa(T))/(*Msp*I(G)/ *Eco*RI_Msp(T))] × 100]. The same internal control DNA sample was used for each pyrosequencing run. Finally, considering complete digestions, GDMR integrates data from nearly six million CCGG sites detected *in silico* on sheep reference genome OAR v3.1.

### Behavioral Traits

Two behavioral tests were used to evaluate sociability for conspecifics and reaction to a human. A complete description of the devices and test procedure used for behavioral measurements are given in Hazard et al. (2016). Briefly, the arena test (AT) aimed to evaluate sociability for conspecifics in three successive phases: (1) attraction for conspecifics with visual contact (Arena test phase 1, AT1), (2) reactivity to social separation from conspecifics by preventing visual contact between the tested lamb and conspecifics (Arena test phase 2, AT2), and (3) attraction for conspecifics in presence of a motionless human standing in front of conspecifics (Arena test phase 3, AT3). The variables used were the frequency of low-pitched bleats (i.e., the lamb bleated with its mouth closed) recorded during AT1 (AT1-LBLEAT), and the frequency of high-pitched bleats (i.e., the lamb bleated with its mouth open) recorded during AT2 (AT2-HBLEAT). Locomotor activity was assessed by measuring the number of zones crossed during AT2 (AT2-LOCOM). Vigilance postures (i.e., animal motionless, head in an upright position, and ears perpendicular to the head) were measured during AT2 (AT2-VIGIL). The time spent in each virtual zone was recorded and the ewe’s proximity score to conspecifics during AT3 (AT3-PROX) was calculated by weighting the time spent in each zone.

The corridor test (CT) aimed to evaluate the reaction to a human. The second phase of the CT was used to evaluate the lamb’s reaction to a walking human. The mean distance between the tested lamb and the walking human was recorded (CT2-DIST).

### Zootechnical Traits

At birth, the type of lamb birthcoat was graded on a scale of one to nine based on composition and structure of the coat, i.e., grade 1, single wooly coat with no halo- or coarse hair, hair length < 10 mm; grade 9, double hairy coat (coarse hair mixed with fine down) > 25 mm in length. Further details on this trait are provided in Allain et al. (2014). Lambs were weighed at birth (birth weight, kg) and weaning (weaning weight, kg). Lambs were also weighed regularly from lambing to weaning to estimate average daily gain. Growth rates (i.e., average daily gain, ADG, in g) were estimated in all lambs from birth to 30, from 30 to 60 and from 60 to 90 days of age (called ADG0-30, ADG30-60, ADG60-90, respectively). In addition, some carcass traits were measured at slaughter only for males, including dressing yield (DY, percentage), conformation score (CONF, score 0–6), compactness (COMP) (i.e., width/length ratio of the carcass, percentage), external fat score (FAT Score, score 0–9), and back fat depth at the 12th rib (FAT depth, mm).

### Statistical Analysis

Analyses of variance were performed to assess the significant fixed effects affecting each measured trait. The tested effects were sex (male or female), born and reared litter size, age of the dam, age and weight of the lambs and year of measurement. The litter size effect was classified according to the number of lambs born and suckled (class 1, ewes lambing and suckling singletons; class 2, ewes lambing twins but suckling only one lamb; class 3, ewes lambing and suckling twins; and class 4, ewes lambing and suckling more than two lambs). The age of the dam effect included ewes that were one, two, and three or more than 3 years old (classes 1, 2, and 3, respectively). The year of measurement effect covered the 5 years over which data were collected. The GLM procedures of the SAS^®^ software (SAS Institute Inc, 1999) were applied to the variables to test the fixed effects and first order interactions. The factors of variation showing significant effect (i.e., *P*< 0.05) on the considered trait were included in subsequent genetic analyses. The fixed effects considered depended on the trait. In addition, for the GDMR trait, we investigated differences between lamb families by testing a family effect (10 classes), nested in the year effect, according to the GLM procedure describe above. This was only used for GDMR because the genetic variability of the other traits included in the present study has already been described (Bibé et al., 2002; Allain et al., 2014; Hazard et al., 2014).

### Genetic Analyses

The (co)variance components of each trait were estimated by restricted maximum likelihood applied to an animal model using ASREML 3.0 software (Gilmour et al., 2009). For each trait, the model included the appropriate fixed effects (i.e., sex, born and reared litter size, age of the dam, year of measurement) and a direct additive genetic effect of the animal considered as random effect. For some traits analyzed, a litter or a dam permanent environmental effect was added as random effect in the model based on present results or the results of previous studies that described an appropriate model for the trait concerned (Bibé et al., 2002; Allain et al., 2014; Hazard et al., 2014). For GDMR, weight and growth traits, the dam was considered as a permanent environmental effect. For birthcoat, the animal’s litter was considered as a permanent environmental effect. The following complete animal mixed model was fitted:

(1) y=X⁢β+Z⁢aa+W⁢cc+e

where *y* is the vector of observations for the trait(s) being analyzed, β is the vector of appropriate fixed effects (sex of lambs, litter size born and suckled, age of the dam and year of measurement), *a* is the vector of random genetic effects and *c* is the vector of permanent environmental effects, when appropriate, with incidence matrices X, Z_*a*_, and W_*c*_, respectively, and e is the vector of residual effects. Univariate analyses were performed to estimate the variance of each trait. Bivariate analyses were performed to estimate the genetic correlations between the GDMR and the other traits using the same model as that used in univariate analyses.

### Genomic Analyses

Genomic analyses were only done for the GDMR trait. Nine of the 10 families were used (i.e., family 10 was not genotyped). Among the 1,047 lambs phenotyped for GDMR, 800 lambs were genotyped (i.e., after filtering an individual call rate ≥ 98%) as well as their nine respective sires using the Illumina OvineSNP50 BeadChip (i.e., 54,241 SNPs). Outlier individuals were removed, and genomic analyses were performed using 775 phenotyped and genotyped lambs. SNP quality was checked as described by Hazard et al. (2014) (i.e., SNP call rates < 97%, a minor allele frequency < 1%, inconsistent for Hardy-Weinberg disequilibrium). Sex chromosomes were not included for analysis. Finally, 40,725 autosomal SNPs were retained for QTL analyses.

Genome wide association analysis was performed of the whole population genotyped using joint analysis considering simultaneously linkage association and linkage disequilibrium (LDLA) to take advantage of both family structure and linkage disequilibrium (Legarra and Fernando, 2009) using QTLMAP software (Gilbert et al., 2008). The LDLA method consisted in interval mapping and LDLA model considered the sire haplotypes effects of the LD model in addition to sire QTL effect. A haplotype size of four SNP was used, and when haplotype frequency was less than 1%, haplotypes were assigned to a rare haplotype group. The chromosome-wise *p*-values were estimated assuming that, conditional on the QTL position, the likelihood ratio test statistics followed a χ^2^-distribution with k degrees of freedom, k being the number of genetic effects (Piepho, 2001). In our study, k was equal to the number of haplotypes plus the number of families for LDLA. Genome-wise *p*-values were obtained by correcting for multiple testing assuming that the number of independent tests was equal to the number of chromosomes analyzed (i.e., 26 independent tests corresponding to the 26 autosomes) (Benjamini and Hochberg, 1995; Knott et al., 1998). Confidence intervals were determined using the “2-LOD drop-off” criterion and assuming 1 LOD = 4.61 LRT (Lynch and Walsh, 1998).

For each QTL, the confidence interval expanded by 2 Mb on each side was used to extract gene annotation by using the Biomart tool from Ensembl release 101 of the sheep reference genome OAR v3.1^1^. Functional annotations for epigenetic modifiers were extracted from dbEM^2^ (Nanda et al., 2016) and from the review about epigenetic modifiers by Feinberg et al. (2016).

## Results

### Phenotypes and Genetic Analyses

Descriptive statistics of GDMR are summarized in Table 1. The mean value of GDMR was 70.71% and the coefficient of variation was 8.40%. Analysis of the gender effect indicated that female lambs had higher GDMR than male lambs. Marked differences were observed between extreme values within a gender. Phenotypic variability of male lambs was higher than female lambs. No significant effects of litter size, age of the dam, age of the lamb and weight (at birth or at weaning) were found, but a significant effect of family (nested in the year effect) was found on GDMR. One family had a low GDMR at 66.60% (family 3) contrasting with four families having a higher GDMR ranging from 71.95 to 74.11% (families 1, 2, 7, and 8). Four families had intermediate GDMR ranging from 68.86 to 70.85% (families 4, 5, 6, 9, and 10). The difference between extreme families reached 1.25 SD. Differences in phenotypic variability were observed between families.

**TABLE 1:** Number of records, mean (± SD), minimum, maximum, quartiles, and least squares means for global DNA methylation rate in the whole population and for each sex and each family.

		**Number of lambs**	**Mean (±SD)**	**Min**	**Max**	**Q1**	**Q3**	***P*-value**	**LSMeans**
Population		1047	70.71 (5.97)	23.08	87.94	68.72	74.24		
Sex	Male	426	68.75 (7.21)	23.08	80.63	66.54	72.60	***	69.12
	Female	621	72.05 (4.47)	40.61	87.94	70.01	74.85		72.17
Family (year)	1	93	73.40 (4.37)	55.82	81.28	71.17	76.81	***	73.05
	2	77	74.73 (3.55)	60.56	80.79	72.82	77.21		74.11
	3	116	66.94 (8.15)	27.04	75.34	65.34	71.46		66.63
	4	91	71.27 (6.7)	23.08	79.85	69.83	75.05		70.85
	5	115	68.95 (4.95)	37.96	77.06	66.24	72.05		68.86
	6	117	69.40 (5.62)	43.39	87.94	66.82	72.18		69.29
	7	101	73.00 (3.89)	55.01	77.89	71.30	75.87		72.57
	8	108	72.20 (3.31)	61.02	78.83	70.52	74.43		71.95
	9	123	69.92 (5.60)	41.62	79.56	68.65	73.03		69.81
	10	106	69.64 (6.73)	28.03	77.22	68.19	73.32		69.33

***P-value < 0.001. Min, minimum; max, maximum; Q1, first quartile; Q3, third quartile; LSMeans, least squares means; Family effect was nested in the year effect.

The estimated variance components of GDMR are listed in Table 2. The estimated heritability for GDMR was moderate (0.20 ± 0.05) and the permanent environment effect of the dam was very low. The additive genetic coefficient of variation for GDMR was also low (3.5%).

**TABLE 2:** Estimates of heritability, repeatability, permanent and residual effects (±S.E.) for the global DNA methylation rate.

		**Component**			**Repeatability (*r*^2^)**	**Total σ*_*p*_*^2^**
	***n***	**Genetic (*h*^2^)**	**Permanent (*m*^2^)**	**Residual (*e*^2^)**		
GDMR	1,047	0.20 ± 0.05	0.02 ± 0.03	0.78 ± 0.05	0.22 ± 0.05	31.3 ± 1.5

*h^2^, m^2^, e^2^, proportion of total phenotypic variance attributed to additive genetic, permanent, and residual effects, respectively, Total σ_*p*_^2^, total phenotypic variance; r^2^ = h^2^ + m^2^; n, number of animals.*

Descriptive statistics and genetic parameters for additional traits recorded in the lambs used in the present study are reported in Table 3. Heritability estimates for behavioral, zootechnical and carcass traits were generally moderate to high (0.20 ± 0.05 to 0.74 ± 0.07). Only low-pitched bleats and conformation score traits showed low heritability. No genetic correlation was found between GDMR and behavioral, birthcoat or zootechnical traits.

**TABLE 3:** Number of records, mean (±SD), estimates of heritability, and permanent environmental effects for behavioral, zootechnical and carcass traits, and genetic correlations between these traits and the global DNA methylation rate.

	***n***	**Mean (±SD)**	***h*^2^**	***p*^2^**	***r*_*g*_**
AT1-LBLEAT	1,047	0.28 (0.28)	0.17 (0.07)	ne	<0.05 (<0.01)
AT2-HBLEAT	1,047	2.91 (1.39)	0.46 (0.07)	ne	0.05 (0.03)
AT2-LOCOM	1,017	19.44 (8.5)	0.26 (0.07)	ne	<0.05 (<0.01)
AT2-VIGIL	1,017	20.68 (9.81)	0.32 (0.07)	ne	<0.05 (<0.01)
AT3-PROX	990	26.26 (16.89)	0.27 (0.06)	ne	<0.05 (<0.01)
CT2-DIST	1,047	5.37 (1.14)	0.20 (0.06)	ne	<0.05 (<0.01)
Birthcoat (score 0–9)	1,032	6.06 (3.38)	0.74 (0.07)	0.15 (0.04)	<0.05 (<0.01)
Birth weight (kg)	1,047	3. 9 (0.8)	0.26 (0.09)	0.27 (0.05)	<0.05 (<0.01)
Weaning weight (kg)	1,043	23.3 (4.2)	0.36 (0.09)	0.20 (0.04)	<0.05 (<0.01)
ADG0-30 (g)	1,047	360.3 (73.04)	0.30 (0.08)	0.24 (0.04)	<0.05 (<0.01)
ADG30-60 (g)	1,047	227.4 (50.5)	0.34 (0.08)	0.06 (0.04)	<0.05 (<0.01)
ADG60-90 (g)	1,046	216.0 (58.0)	0.32 (0.07)	0.10 (0.04)	<0.05 (<0.01)
DY (%)	386	42.07 (2.19)	0.41 (0.15)	ne	<0.05 (<0.01)
CONF (score 0–6)	390	3.44 (0.8)	0.09 (0.08)	ne	<0.05 (<0.01)
COMP (%)	388	32.26 (1.46)	0.58 (0.20)	ne	<0.05 (<0.01)
FAT score (score 0–9)	390	5.07 (1.39)	0.22 (0.12)	ne	<0.05 (<0.01)
FAT depth (mm)	389	1.87 (1.36)	0.36 (0.13)	ne	<0.05 (<0.01)

*AT1/2/3, arena test phase 1/2/3; CT2, corridor test phase2; LBLEAT, low bleats; HBLEAT, high bleats; LOCOM, locomotion; VIGIL, vigilance behavior; PROX, proximity score; DIST, mean distance between tested animal and a human; ADG, average daily gain; DY, dressing yield; CONF, conformation score; COMP, compactness. h^2^, p^2^, proportion of total phenotypic variance attributed to additive genetic or permanent environmental effects, respectively, r_*g*_, genetic correlations; standard errors are in parentheses; ne, not estimated.*

### Genomic Analyses

**FIGURE 1:**
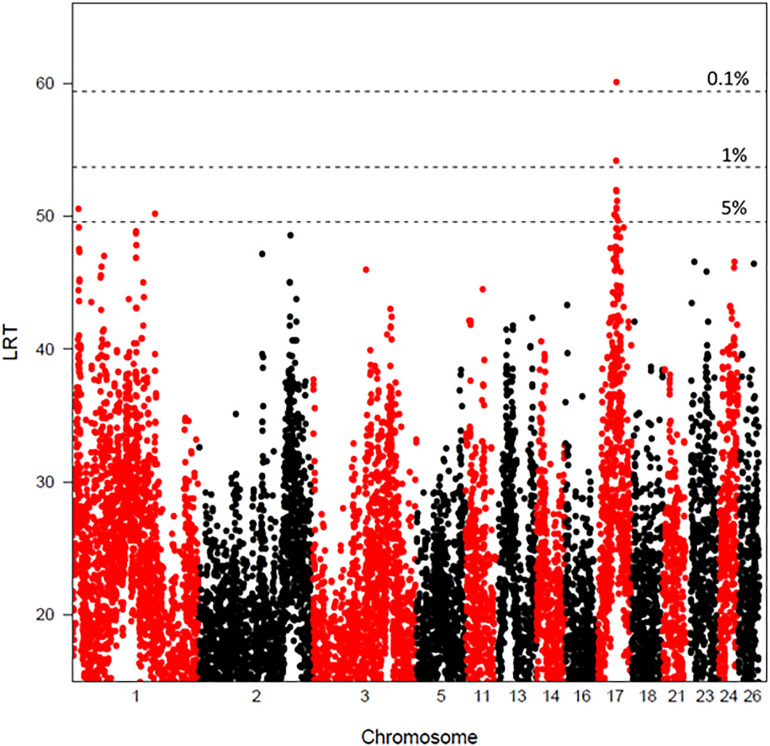
Chromosome plots of the likelihood ratio test values obtained for global DNA methylation rate by LDLA analysis. The likelihood ratio test (LRT) is plotted against SNP haplotype positions (four consecutive SNPs) for chromosomes 1, 2, 3, 5, 11, 13, 14, 16, 17, 18, 21, 23, 24, and 26. The horizontal lines indicate the 5, 1, and 0.1% genome wide thresholds. One percent chromosome wide thresholds were OAR3, 47.6; OAR5, 37.4; OAR13, 42.6; OAR14, 35.9; OAR16, 38.7; OAR17, 42.3; OAR18, 40.6; OAR21, 37.3; OAR23, 45.7; OAR24, 42.8, and OAR26, 47.1. 5% chromosome wide thresholds were OAR1, 45.5; OAR2, 45.0; OAR11, 41.3; OAR13, 38.3; OAR24, 38.4.

The significant QTLs found using LDLA and reaching genome-wide (GW) or chromosome-wide (CW) significance thresholds are reported in Table 4 and Figure 1. Four haplotype-trait associations reached the GW significance thresholds and 16 haplotype-trait associations reached the 1% CW significance threshold. Eight significant associations reaching the 5% CW threshold were also detected. Associations found in LDLA mapped to 14 chromosomes. The four associations that reached the GW threshold of significance localized on chromosomes 1 (OAR1, 13.20 and 179.50 Mb) and 17 (OAR17, 40.14 and 55.54 Mb). Associations reaching the 1% CW significance threshold were mapped on chromosomes OAR3, 5, 13, 14, 16, 17, 18, 21, 23, 24, and 26. Effects of QTLs found in LDLA ranged from 0.68 to 3.20% of the total phenotypic variance of GDMR.

**TABLE 4:** List of QTLs detected in joint analysis (linkage disequilibrium linkage analysis) associated with the global DNA methylation rate.

**Chr.**	**Significance**	**level^a^**	**QTL effect (%)^b^**	**Confidence interval (Mb)**	**Flanking markers^c^**		**Annotated protein coding genes within confidence interval**	**Epigenetic modifier candidate**
1	**GW**	*	2.48	13.1–13.4	OAR1_13033760.1	s62110.1	RRAGC, GJA9, RHDB	
1	CW	*	2.75	69.3–69.5	OAR1_74015424.1	OAR1_74065628.1	MTF2	
1	CW	*	3.0	138.0–138.3	OAR1_149183890.1	OAR1_149346875.1	–	
1	**GW**	*	2.4	179.4–179.6	OAR1_193623565.1	OAR1_193664187.1	–	
2	CW	*	1.44	199.7–199.9	OAR2_211501156.1	OAR2_211573792.1	–	
3	CW	**	1.0	114.5–114.7	OAR3_122117752.1	OAR3_122207054.1	–	
5	CW	**	0.83	96.6–96.9	OAR5_105186382.1	OAR5_105374983.1	–	CHD1
11	CW	*	2.95	10.5–10.9	s74938.1	OAR11_10495137.1	MAD13, INTS2, BRIP1	
11	CW	*	0.85	37.4–37.6	OAR11_39854191.1	OAR11_39948154.1	HOXB1/2/3/4/5/6/7/9	KAT7
13	CW	*	2.9	17.8–18.5	OAR13_20537279.1	OAR13_20578061.1	PARD3	
13	CW	*	2.7	32.1–32.3	OAR13_35499997.1	OAR13_35648372.1	NSUN6, EPC1	
13	CW	**	1.17	74.5–74.7	s65442.1	s62908.1	CDH22, SLC35C2, ELMO2	NCOA3
14	CW	**	2.45	7.7–7.9	s16689.1	OAR14_8081413.1	CMIP, PLCG2, SNORA70	
14	CW	**	2.06	10.7–10.9	s26487.1	s43948.1	–	
14	CW	**	2.77	14.8–15.2	OAR14_15219778.1	OAR14_15268863.1	GTP2, DNAJA2, NETO2, ITFG1	
14	CW	**	2.85	17.5–18.3	s22016.1	s31525.1	ZNF423, CNEP1R1, HEATR3, TENT4B, ADCY7, BRD7	
16	CW	**	2.38	4.5–4.8	OAR16_4698169.1	OAR16_4737110_X.1	DUSP1, ERGIC1, RPL26L1, ATP6V0E1	
17	CW	**	2.7	26.7–26.9	OAR17_29456634.1	s52728.1	–	
17	**GW**	***	2.71	40.0–40.2	OAR17_43310387.1	OAR17_43356226.1	PPID, ETFDH, C4orf46, RXFP1	
17	**GW**	*	2.8	55.4–55.6	OAR17_60522910.1	s57641.1	CCDC60	
18	CW	**	1.68	6.6–6.8	OAR18_6256173.1	s37275.1	TTC23, SYNM	
21	CW	**	0.80	3.7–4.1	OAR21_4678890.1	s05427.1	–	
23	CW	**	0.68	8.3–8.5	OAR23_9159961.1	OAR23_9204273.1	–	
23	CW	**	2.7	34.4–34.6	OAR23_36433223.1	s07796.1	GATA6	
24	CW	*	2.5	7.4–7.7	OAR24_8719574.1	s03253.1	TMEM114, METTL22, ABAT, TMEM186, PMM2, CARHSP1	
24	CW	**	3.2	23.6–24.0	s12637.1	OAR24_26210500.1	–	KDM8
24	CW	**	1.08	32.6–33.0	s64882.1	s17643.1	RCC1L, NCF1, GTF2IRD1, CLIP2	
26	CW	**	2.0	33.4–33.6	OAR26_38152631.1	OAR26_38251830.1	ADAM18, IDO1, IDO2	KAT6A

Significant QTLs reaching the chromosome-wide or the genome-wide thresholds are listed. ^a^*p < 5%; **p < 1%; ***p < 0.1%. ^*b*^Percentage of phenotypic variance explained by the haplotype with the most effect (absolute value). ^*c*^SNPs flanking the QTL with significant association. CW, chromosome wide; GW, genome wide; Position and genes are based on the reference genome Oar_v3.1.

By crossing information from sheep reference genome annotation (OAR v3.1), the database of epigenetic modifiers (dbEM, (Nanda et al., 2016) and those reported by Feinberg et al. (2016), we highlighted *CHD1, NCO3A, KDM8, KAT7*, and *KATA6* as the most obvious candidate genes from each QTL (+2 Mb each side of the confidence interval), likely to play a role in the GDMR variation (Table 4).

## Discussion

The global DNA methylation rate measured by LUMA reached 70% in sheep blood. This result is consistent with global DNA methylation percentages reported in human blood analyzed by LUMA (for a review, see Soriano-Tarraga et al., 2013). The value of GDMR in the present study is also in the range of calculated DNA methylation either using the global CpG methylation rate (50–55%) measured by reduced representation bisulfite sequencing (RRBS) in sheep muscle (Couldrey et al., 2014) or the total methyl content measured by high performance liquid chromatography reported in somatic tissues (80%) in humans (Ehrlich et al., 1982). The large range observed for DNA methylation rate across the genome is likely due to the methods used for quantification of DNA methylation and/or the tissues studied. Indeed, in their review, Soriano-Tarraga et al. (2013) reported that variation in global DNA methylation in human blood analyzed by LUMA ranged from 52 to 78% depending on the DNA isolation method used. In addition, Doherty and Couldrey (2014) reported that RRBS libraries are biased because they contain promoter regions which have high CG content known to be largely devoid of DNA methylation. Consequently, RRBS libraries are expected to display lower methylation on average across genome than unbiased libraries.

Variability was observed for the GDMR phenotype recorded in this study. Part of this variability originated from differences between male and female lambs. Sex differences in the methylome have already been reported in humans. In human liver, females displayed higher average DNA methylation in the X-chromosome whereas males presented higher DNA methylation in autosomes in this tissue (García-Calzón et al., 2018). Genome-wide sex differences in locus-specific DNA methylation across autosomes have also been reported in other human tissues such as blood (Singmann et al., 2015). In the present study, the positions of DNA methylation across genome were unknown, so we are unable to conclude whether gender difference in DNA methylation found in lambs was of similar magnitude on the X-chromosome and autosomes. In addition, it should be kept in mind that we investigated DNA methylation in young lambs (4 months of age), not in adult sheep. A study investigating gender differences in DNA methylation in human blood at birth reported that CpG located on autosomes, as well as DMR, were hypermethylated in girls compared to boys (Yousefi et al., 2015). Because gender differences in DNA methylation across autosomes in human blood varied between newborns and adults, we cannot exclude that a sex by age interaction could affect DNA methylation in sheep blood.

We hypothesized that GDMR has a genetic component in sheep. The variance component decomposition analysis of GDMR revealed moderate heritability (20%). To our knowledge, this is the first heritability estimate for GDMR in livestock species. Estimation of heritability of DNA methylation levels in human whole blood at thousands of sites has shown that heritability estimates vary across the genome but is on average 20%, consistently with our estimate in sheep blood (van Dongen et al., 2016). Additional studies in humans also indicated that the average twin based heritability of DNA methylation across genome-wide CpGs varied between 5 and 19% depending on the tissue (i.e., *h*^2^ = 5, 7, and 12% in placenta, human umbilical vascular endothelial cells and cord blood mononuclear cells from newborns twins, respectively, *h*^2^ = 18% in peripheral blood lymphocytes from adolescent twins [Gordon et al., 2012; McRae et al., 2014]). Interestingly, Shah et al. (2014) reported that DNA methylation in whole blood remained stable over the human lifetime, as indicated by the high correlation (0.68) between DNA methylation repeatability in older people (median age 70–90) and heritability estimated in teenagers.

Genetic relationships between GDMR and production or adaptive traits were also investigated in the present study. We did not find any relationship between GDMR and production or adaptive traits in domestic sheep. Our results contrasted with the negative correlation between total DNA methylation level and daily growth rate in fish embryos (Ou et al., 2019). To our knowledge, no published works provide evidence of a relationship between GDMR in blood and the specific adaptive traits used in the present study (social behaviors and birthcoat). Some relationships would be expected, since several studies suggested epigenetic influence on adaptive processes in mammals (Massicotte et al., 2011; Lea et al., 2016). Nevertheless, only a few adaptive traits were explored in this study and we cannot exclude the possibility that such relationships exist for other adaptive traits or in older animals. Additionally, regardless of the absence of relationships between GDMR and adaptive traits in our study, we cannot exclude, by considering individually DNA methylation sites, to find epi-loci associated with production or adaptive traits investigated in the present study. Indeed, epi-loci have previously been reported to be associated with animal behaviors (Champagne and Curley, 2009; Roth, 2013; Verhulst et al., 2016; Seebacher and Krause, 2019).

Even if the greater attention paid to epigenetics in farm animals has led to an increasing number of GWA epigenetic studies, genomic studies of DNA methylation rate had remained to be performed. In our study, we were particularly interested in detecting QTLs for GDMR in domestic sheep. The QTL analysis for GDMR resulted in mapping 28 QTLs on 14 chromosomes, with four regions (on OAR1 and OAR17) reaching the genome wide level of significance. Each detected QTL explained less than 3% of the phenotypic variance. Our experimental design enabled to reach a power of 90% for the detection of a QTL explaining 8% of the phenotypic variance while the power was 20% for a QTL that explains 1% of phenotypic variance (Luo, 1998). Consequently, the undetected QTLs likely had a limited effect on GDMR. Thus, the low proportion of variance explained by each QTL supports the hypothesis that GDMR in domestic sheep is under polygenic influence and unlikely under the control of a major gene. Nevertheless, several QTLs mapped in the present study could probably act in combination to account for substantial genetic variation of GDMR.

To better understand the underlying molecular mechanisms associated with these QTLs, we looked for possible overlapping between the location of the identified QTLs (confidence interval enlarged by 2 Mb each side) and the location of annotated genes relevant to epigenetics. Despite the large number of genes located close to the QTL regions identified, only few genes seem relevant as epigenetic modifiers through their action on methylation or acetylation processes on DNA or histones. Indeed, by crossing the list of positional genes in enlarged QTL regions with that of known epigenetic modifiers either from the dbEM database (Nanda et al., 2016) or those listed in Feinberg et al. (2016), only *CHD1, KDM8, NCO3A, KAT7*, and *KATA6* appeared as the most obvious candidate genes. *CHD1* gene encodes the chromodomain-helicase-DNA-binding protein 1 (OAR5: 95.4 Mb), that functions as substrate recognition component of the transcription regulatory histone acetylation complex SAGA. Mutations in *CHD1* are frequently associated with prostate cancers (Berger et al., 2011). Functional analyses have shown that CHD1 was involved in transient DNA methylation and several loci were hypermethylated in *CHD1* deleted strains of Neurospora crassa (Belden et al., 2011). KDM8 (Lysin demethylase 8 or Jumonji C domain-containing demethylase 5, JDMD5; OAR24: 24.8 Mb) is a histone H3 demethylase with specificity for Lys-36. This demethylase promotes homologous recombination (Amendola et al., 2017), cell proliferation (Hsia et al., 2010) and protects from nerve demyelination (Fuhrmann et al., 2018). Interestingly, a second gene encoding an important histone lysine demethylase, *KDM2B* (Lysine-specific demethylase 2B, OAR17: 53.23 Mb), was found close to one of the enlarged QTL regions (OAR17: 55.4–55.6 Mb). In this region, we also identified *SETD1B* gene (Histone-lysine N-methyltransferase SETD1B, 53.06 Mb) which encodes a component of a histone methyltransferase complex that specifically methylates Lys-4 of histone H3 and is responsible for the epigenetic control of chromatin structure and gene expression. A specific hypermethylation signature was associated with loss of function mutations in the *SETD1B* gene (Krzyzewska et al., 2019). The *NCOA3* gene (nuclear receptor coactivator 3; OAR13: 32.0 Mb) encodes a nuclear receptor coactivator with histone acetyltransferase activity. It particularly interacts with other transcriptional activators such p300/CBP-associated factor and CREB binding protein (CREBBP) as part of a multi-subunit coactivation complex. The *CREBBP* gene, also located near a significant QTL position (OAR 24: 3.1 Mb) has been recently reported to be involved in the crosstalk between DNA methylation and histone acetylation (Zhang et al., 2020). Finally, two lysine acetyltransferase coding genes of the MYST family, *KAT6A* (lysine acetyltransferase 6A, also named *MOZ*, OAR26: 35.3 Mb) and *KAT7* (lysine acetyltransferase 7, also named *HBO1*, OAR11: 36.4 Mb) has been reported to play a key role in acetylation of lysine residues in histone H3 and/or H4 (Kitabayashi et al., 2001). Interestingly, KDM8, NCOA3 and KAT7 act as androgen or estrogen receptor co-regulators (Sharma et al., 2000; Vija et al., 2013; Wagner et al., 2013; Wang et al., 2019). Polymorphisms that alter the function of these genes could explain that female lambs had higher GDMR than male lambs.

The level and state of histone acetylation regulated by histone acetyl transferase enzymes are widely reported to be an epigenetic regulation of gene expression (Kouzarides, 2007). Therefore, we cannot exclude the possibility that global DNA methylation in sheep is associated with histone acetylation, since relationships between DNA methylation and histone de-acetylation have already been described (Geiman and Robertson, 2002; Vaissière et al., 2008). Several other coding genes, located close to QTLs we identified, have been reported to be involved in the regulation of transcription or regulation of chromatin architecture. Although no scientific evidence has suggested their involvement in DNA methylation to date, these genes could mediate this process through their active role in the coregulatory complexes that associate epigenetic modifiers to modulate transcription.

## Conclusion

Inter-individual variability in global DNA methylation rate in blood has an additive genetic component in sheep and heritability was moderate (*h*^2^ = 0.20 ± 0.05). This opens the way for a possible genetic selection of this trait and create experimental divergent lines to investigate further the relationship between GDMR and adaptive traits as already evidenced in plants (Johannes et al., 2009; Kooke and Keurentjes, 2015). Moreover, this work reported the first SNP-based QTL detection study for GDMR as a quantitative trait in livestock species. The evidence of 28 QTLs associated with blood GDMR, each explaining a small proportion of the phenotypic variance (between 1 and 3%), most likely indicated a polygenic determinism of this trait. The further identification of genes and their polymorphisms underlying the global DNA methylation rate assessed in our study will paves the way for a deeper understanding the genetic component of such a trait.

## Data Availability Statement

The raw data supporting the conclusions of this article will be made available by the authors, without undue reservation.

## Ethics Statement

The animal study was reviewed and approved by the Local ethics committee SCIENCE ET SANTE ANIMALES N°115 (Ecole Nationale Vétérinaire de Toulouse).

## Author Contributions

DH, LD, CM-R, and SF conceived and designed the genetic experimental design and were involved in planning the study. FP-P was in charge of the phenotyping of GDMR. DH performed statistical and genetic analyses. DH, LD, SF, FP-P, and CM-R analyzed and interpreted the results. DH drafted the manuscript. LD, SF, and CM-R helped in writing, reviewing, and editing the final manuscript. All authors have read and approved the final manuscript.

## Conflict of Interest

The authors declare that the research was conducted in the absence of any commercial or financial relationships that could be construed as a potential conflict of interest.

## Footnotes

1 http://www.ensembl.org

2 http://crdd.osdd.net/raghava/dbem
